# Quantitative Sensory Testing Identifies Altered Thermal and Pain Processing in Trigeminal Neuralgia

**DOI:** 10.1111/ene.70553

**Published:** 2026-06-01

**Authors:** Gianfranco De Stefano, Daniel Litewczuk, Eleonora Galosi, Giuseppe Di Pietro, Pietro Falco, Caterina Leone, Jibril Osman‐Farah, Deepti Bhargava, Francis O'Neill, Bernhard Frank, Colby T. Joncas, Raymond F. Sekula, Giulia Di Stefano, Andrea Truini

**Affiliations:** ^1^ Department of Human Neuroscience Sapienza University Rome Italy; ^2^ The Walton Centre NHS Foundation Trust Liverpool UK; ^3^ The Pain Research Institute University of Liverpool Liverpool UK; ^4^ Department of Neurological Surgery Columbia University Medical Center New York New York USA; ^5^ Department of Neurology, Columbia University Vagelos College of Physicians and Surgeons New York New York USA

**Keywords:** central sensitization, neuropathic pain, quantitative sensory testing, sensory processing, trigeminal neuralgia

## Abstract

**Introduction:**

This cross‐sectional study aims to assess somatosensory function in patients with trigeminal neuralgia (TN) using quantitative sensory testing (QST) and QST‐based sensory phenotyping, and to investigate their relationship with clinical findings, including etiology (classical and idiopathic TN), type of pain (paroxysmal pain only and concomitant continuous pain), and response to pharmacological treatment.

**Methods:**

Sixty‐five patients with classical or idiopathic TN underwent QST. Sensory alterations were quantified using *z*‐scores derived from normative data, and sensory profiles were identified using a validated algorithm. Clinical characteristics, presence of concomitant continuous pain, and response to first‐line sodium channel blockers were systematically recorded through a structured questionnaire.

**Results:**

QST showed bilateral thermal hypoesthesia and pain hyperesthesia in the whole patient population. Cold detection threshold (CDT) was significantly reduced on the affected side compared to the unaffected side in patients with Classical TN. Idiopathic TN was associated with bilaterally elevated wind‐up ratio. Patients with purely paroxysmal pain showed a bilateral gain of function in heat pain thresholds. Sensory phenotyping identified mechanical hyperalgesia (49%) and thermal hyperalgesia (35%) as predominant phenotypes. A sensory loss phenotype was significantly associated with poor response to sodium‐channel blockers.

**Discussion:**

TN is characterized by subtle and complex bilateral sensory alterations, potentially suggesting central modification of sensory processing. The finding of asymmetrical CDT reduction may indicate peripheral Aδ‐fiber dysfunction. Sensory profiling may identify subgroups with distinctive treatment responses, with sodium channel blockers being less effective in sensory loss phenotype.

## Graphical abstract

DFNS‐based quantitative sensory testing (QST) and sensory phenotyping were performed on the most affected trigeminal division in 65 patients with trigeminal neuralgia (TN). In the overall cohort, QST revealed bilateral thermal hypoesthesia and bilateral pain hyperalgesia, with mechanical and thermal hyperalgesia being the most frequent sensory phenotypes. Classical TN was associated with reduced CDT on the affected side, idiopathic TN with bilateral increases in WUR, purely paroxysmal TN with bilateral increases in HPT, and a sensory loss phenotype was associated with poor response to sodium‐channel blocker medications. Created in https://BioRender.com. CDT, cold detection threshold; CPT, cold pain threshold; DMA, dynamical mechanical allodynia; HPT, heat pain threshold; MDT, mechanical detection threshold; MPS, mechanical pain sensitivity; MPT, mechanical pain threshold; PHS, paradoxical heat sensations; PPT, pressure pain threshold; TSL, thermal sensory limen; VDT, vibration detection threshold; WDT, warm detection threshold; WUR, wind‐up ratio.

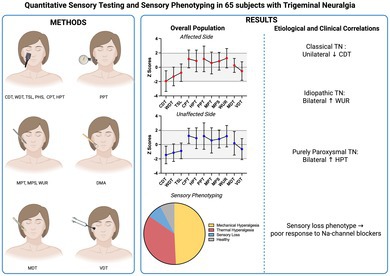

## 1 Introduction

Trigeminal neuralgia (TN) is a specific neuropathic pain condition characterized by sudden, sharp, and brief episodes of unilateral facial pain, often described as electric shock‐like, evoked by tactile innocuous stimulation of trigger zones [1, 2]. In addition to this characteristic paroxysmal pain, up to 50% of patients report an additional, more prolonged pain—referred to as concomitant continuous pain—commonly described as dull or burning in nature [3, 4]. TN is classified based on its etiology into secondary TN, most commonly related to tumor or multiple sclerosis, classical TN, associated with neurovascular compression causing anatomical changes of the trigeminal root, and idiopathic TN, of unknown etiology [5, 6].

According to the diagnostic criteria developed by the Neuropathic Pain Special Interest Group of the International Association for the study of pain, sensory abnormalities within the area of pain are required to diagnose probable neuropathic pain [7]. However, detecting such abnormalities in the facial region is often difficult and time consuming, and notably, they are not required for diagnosing trigeminal neuralgia (TN)—a unique exception among neuropathic disorders [8]. The limited availability of robust, reproducible methods to assess somatosensory function in TN remains a significant gap in understanding, especially since neurophysiological tests (e.g., trigeminal reflexes) usually show minimal findings [9].

Quantitative sensory testing (QST) is a standardized psychophysical method used to assess the function of all classes of sensory afferents. It supports routine clinical examination by quantifying sensory abnormalities and identifying both negative and positive signs of abnormal somatosensory processing [10]. In addition, QST enables the stratification of patients into distinct sensory phenotypes, which may help predict treatment response in neuropathic pain conditions [11]. As such, QST could deepen our understanding of trigeminal sensory abnormalities and phenotypes in TN, with potential implications for improving diagnosis and treatment.

In this cross‐sectional study, we aimed to identify sensory abnormalities and phenotypes in patients with TN using QST and investigate how these abnormalities correlate with clinical findings, including etiology (classical and idiopathic TN), type of pain (paroxysmal pain only and concomitant continuous pain), and response to pharmacological treatment.

## 2 Methods

### 2.1 Study Population

We prospectively screened consecutive patients attending the Peripheral Neuropathy and Neuropathic Pain Unit at the Human Neuroscience Department of Sapienza University, Rome, from November 2020 to November 2024, and patients visiting the Joint Facial Pain Clinic at The Walton Centre NHS Foundation Trust, Liverpool, UK, from August 2021 to June 2022. The study protocol was approved by both the institutional review boards. Written informed consent was obtained from each participant.

Inclusion criteria were a definite diagnosis of TN according with the ICHD‐3 2018 and ICOP‐1 2020 criteria [1, 2] and a 3T MRI scan, including high resolution CISS sequences detailing the posterior fossa, aimed at the etiological classification of TN [5]. Final diagnoses considered for inclusion were classical TN (when MRI identified a neurovascular compression with major morphological changes, like atrophy or dislocation, of the affected trigeminal root) and idiopathic TN (when MRI identified no vascular contact or vascular contact without major changes). The etiology of classical or idiopathic TN was independently confirmed by at least two clinicians (Rome: AT, GDiS; Liverpool: FON, BF, JOF, DB). Exclusion criteria were a diagnosis of secondary TN, a diagnosis of orofacial pain other than TN, bilateral facial pain, a diagnosis of other neurological or psychiatric diseases (including major depression), any sensory abnormalities in the face identified at bedside clinical assessment, previous surgery for TN, cognitive disturbances, or communication barriers.

Each subject underwent a structured questionnaire published elsewhere [9] to systematically collect demographic and clinical information. In particular, the interview was aimed to identify the division most affected by pain, the presence of concomitant continuous pain and the ongoing pharmacological treatment. Patients were also asked to rate the global pain relief since the start of their current medications, on a scale from 0 to 100. TN is typically characterized by a marked response to sodium channel blockers such as carbamazepine and oxcarbazepine [12], which are considered first‐line treatments. In contrast, the response to other medications is generally poor. Therefore, we limited our analysis of treatment response to these two drugs, classifying patients with a reported effectiveness rating of 50 or less as non‐responders.

### 2.2 Quantitative Sensory Testing

QST was performed in all patients by trained examiners following the standardized protocol of the German Research Network on Neuropathic Pain (DFNS) [10]. We examined the affected division on both sides. For patients with more than one affected division, we assessed the division the patient reported as most affected.

We collected the following parameters: cold detection threshold (CDT), warm detection threshold (WDT), thermal sensory limen (TSL) and the number of paradoxical heat sensations (PHS), cold pain threshold (CPT), heat pain threshold (HPT), mechanical detection threshold (MDT), mechanical pain threshold (MPT), mechanical pain sensitivity (MPS), dynamic mechanical allodynia (DMA), wind‐up ratio (WUR), pressure pain threshold (PPT), and vibration detection threshold (VDT).

For thermal and thermal pain thresholds (CDT, WDT, CPT, HPT) we used a thermode (16 × 16 mm ATS, PATHWAY, Medoc, Ramat Yishai, Israel). The baseline temperature of 32°C reached the target temperature at a ramp rate of 1°C/s. Patients were instructed to push a button as soon as they perceived cold/warm/cold pain/heat pain, respectively. Each test was repeated three consecutive times, and the average among the three measures was considered. Thermal sensory limen was determined by alternately changing the temperature of the thermode starting from 32°C. Patients pushed a button to indicate when a change in temperature was felt. The number of paradoxical heat sensations was also recorded.

Mechanical Detection Threshold (MDT) was evaluated with a standardized set of modified von Frey hairs (Optihair2‐Set, Marstock Nervtest, Germany) that exerted forces between 0.25 and 512 mN. The final threshold was the geometric mean of five series of ascending and descending stimulus intensities.

MPT was measured using “Pinprick” equipment, MRC Systems GmbH‐Medizintechnische System stimulators (flat contact area of 0.2‐mm diameter; forces between 8 and 512 mN). The final threshold was the geometric mean of five series of ascending and descending stimulus intensities. We calculated MPS using the same pinprick equipment. Each pinprick was applied five times in a pseudorandom fashion and rated by the patient on a numerical rating scale (NRS) ranging from 0 (no pain) to 100 (maximum imaginable pain). The final value was the geometric mean of the 35 ratings.

DMA was calculated as the geometric mean of the ratings of 15 tactile stimuli intermingled with the pinprick stimuli in a pseudorandom fashion.

Using the pinprick equipment, we investigated wind‐up ratio using a series of 10 repetitive 128‐mN pinprick stimuli applied at 1 Hz rate. We calculated the ratio between the pain NRS (range 0–100) for the series and the NRS for a single stimulus at the same intensity. The test was repeated 5 times, and the WUR was considered as the arithmetic mean among the 5 ratios.

VDT was performed with a Rydel‐Seiffer tuning fork (128 Hz, 8/8 scale) over a bony prominence of the skull. The semiquantitative threshold for disappearing sensation was considered. Vibration detection threshold was determined as the arithmetic mean of three series of these thresholds.

We measured PPT using a pressure algometer with a contact area 1 cm^2^ (Wagner Instruments, USA), that was applied above the masseter muscle in three series of slowly increasing stimulus intensities. The threshold was then determined as the arithmetic mean of the three series (in kPa).

We then used a published large normative dataset for the face as control, according to the sex and age of each subject [13]. Using the log‐transformed raw values for each QST variable, a *z*‐score sensory profile was calculated using the formula *Z* = (*x* − *μ*)/*σ*, where *x* stands for the value calculated in the specific subject, *μ* the mean value and *σ* the standard deviation of the normative dataset population [13]. Negative *z*‐scores indicated loss of perception, whereas positive *z*‐scores indicated gain of perception. Values exceeding 1.96 SD were considered abnormal.

### 2.3 Sensory Phenotyping

For each participant, we applied a previously published algorithm [11] to calculate the probability of their individual sensory profile to be assigned into one of four characteristic QST phenotypes: (1) “sensory loss,” characterized by reduced sensitivity to both thermal and mechanical stimuli; (2) “thermal hyperalgesia,” presenting with intact sensory perception but increased sensitivity to heat or touch; (3) “mechanical hyperalgesia,” manifesting with diminished thermal detection combined with heightened mechanical sensitivity; and (4) “healthy sensory profile,” defined by a pattern largely comparable to that of healthy individuals. Phenotype assignment was based on a deterministic approach, whereby each patient was categorized according to the phenotype with the highest calculated probability.

### 2.4 Statistical Analysis

We calculated the sample size using the formula for estimating a population mean: *n* = (*Z* × SD/*E*)^2^. Given the limited availability of standard deviation (SD) values for *z*‐normalized QST parameters in previous studies, we adopted a conservative approach by using the highest reported standard deviation from normative data, equal to 1.02 [10, 13]. With a desired margin of error (*E*) of 0.25 and a 95% confidence level (*Z* = 1.96), the resulting minimum sample size was 64 participants.

In the manuscript, continuous variables are presented as mean ± SD (standard deviation) and categorical variables are expressed as frequencies.

For continuous variables, normality of the data distribution was assessed using Shapiro–Wilk test. First, we compared the *z*‐scores in the affected and not affected side of the face using paired Student's *t*‐test or Wilcoxon Signed‐Rank test as appropriate. Multiple comparisons were controlled by applying a Bonferroni correction within each QST sensory domain (thermal detection, thermal pain, mechanical pain, mechanical detection), given the high correlation among parameters within the same domain [14]. Then, we analyzed the interaction between the affected side, the etiology (classical or idiopathic TN) and the type of pain (with or without concomitant continuous pain) using repeated measure two‐way ANOVA. When a significant main effect or interaction was detected, we conducted post hoc analyses using Bonferroni‐corrected multiple comparisons. Categorical variables were compared between groups using Fisher's exact test. Correlation between QST *z*‐scores on both sides and disease duration was explored using Spearman coefficient. Differences in affected divisions, medication use and sensory phenotypes distribution among groups were evaluated using Fisher's exact test.

A *p*‐value of < 0.05 after Bonferroni's correction for multiple comparisons was considered statistically significant. The statistical analysis was conducted using GraphPad 8.0 (La Jolla, CA, USA).

## 3 Results

### 3.1 Demographic and Clinical Characteristics

During the study enrolment timeframe, we prospectively screened 146 subjects in Rome and 38 subjects in Liverpool. Collectively, 24 patients were excluded because the diagnosis of TN was not confirmed by study investigators, 21 due to a diagnosis of secondary TN. Of the remaining 139 patients with a diagnosis of classical or idiopathic TN, 47 were excluded due to previous surgery for TN, nine patients were excluded because of medical contraindications to perform MRI and 18 patients refused or were not compliant to QST examination, two of whom discontinued the procedure because they experienced TN pain during the examination.

A total of 65 patients (47 women) were included in the study, with a mean age of 66.51 ± 11.85 years and a mean disease duration of 7.6 ± 7.1 years. Seven patients reported pain predominantly affecting V1 (11%), 39 mainly involving V2 (60%) and 19 V3 (29%). MRI scans showed neurovascular compression leading to atrophy or dislocation of the trigeminal nerve root in 41 patients, who were subsequently diagnosed with classical TN. In contrast, 24 patients (37%) were diagnosed with idiopathic TN, as no neurovascular contact or only contact without major anatomical changes was observed. Of the 65 patients included in the study, 35 were affected by purely paroxysmal pain (54%, 24 with classical and 11 with idiopathic TN), while 30 also reported concomitant continuous pain (46%, 17 with classical and 13 with idiopathic TN). Forty patients were on therapeutic doses of sodium‐channel blockers at time of enrolment (62%), while 10 were on gabapentinoids monotherapy and 15 were in remission and did not assume any therapy. Ten of the forty patients on sodium channel‐blockers (25%) reported an unsatisfactory global pain relief (i.e., they had a pain relief of 50% or less) with their current treatment (Table 1).

**TABLE 1:** Demographic and clinical characteristics of the patient cohort.

Age	66.51 ± 11.85 years
Sex	47 females (72%) 18 males (28%)
Disease duration	7.6 ± 7.1 years
Affected divisions	7 V1 (11%) 39 V2 (60%) 19 V3 (29%)
Type of pain	35 with purely paroxysmal (54%) 30 with concomitant continuous pain (46%)
Etiology	41 classical (63%) 24 idiopathic (37%)
Current medications	40 under sodium channel blockers (62%) 25 with other medications or in remission phase (38%)
Response to sodium channel blockers	30 responder (75%) 10 non‐responder (25%)

No significant differences were observed between patients with classical and idiopathic TN regarding affected division or medication use, nor between patients with purely paroxysmal pain and those with concomitant continuous pain.

None of the included patients experienced TN pain at the time of the examination.

### 3.2 Quantitative Sensory Testing

**FIGURE 1:**
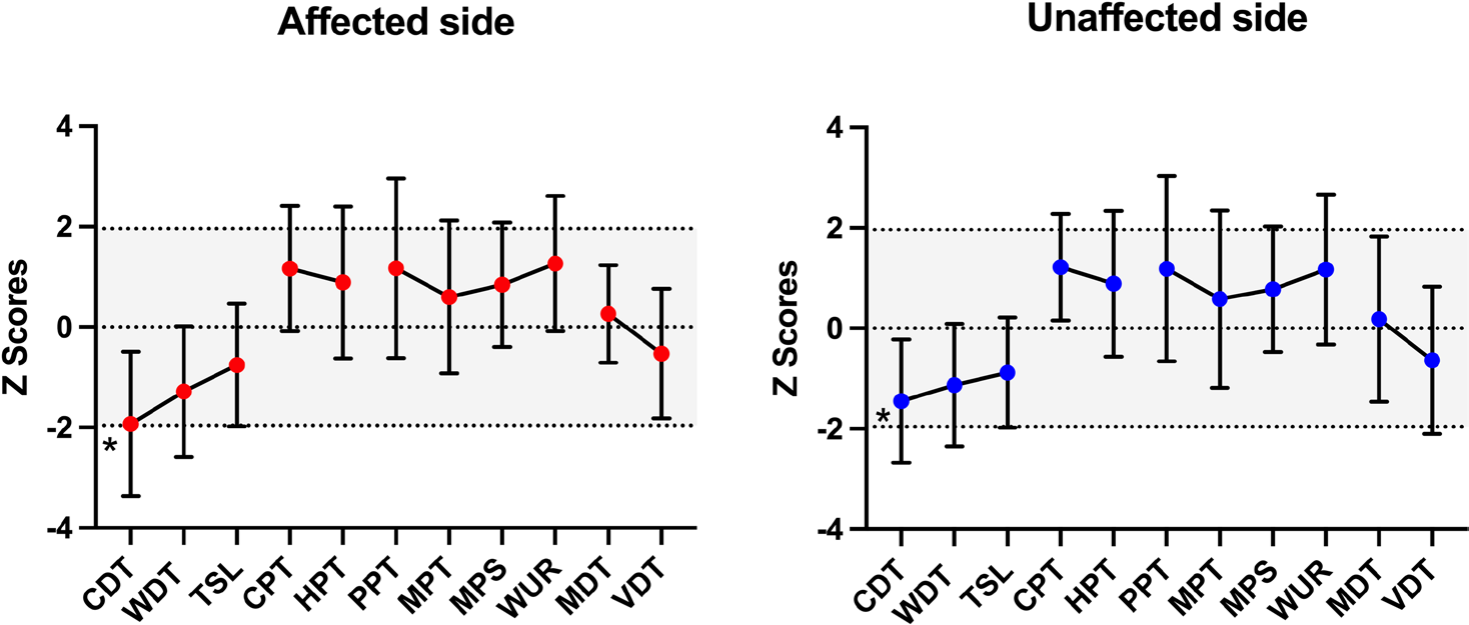
Graphical depiction of the mean *z*‐scores for 11 items of the DFNS protocol of quantitative sensory testing in 65 patients with trigeminal neuralgia, on the affected (left) and not affected (right) side of the face. CDT, cold detection threshold; CPT, cold pain threshold; HPT, heat pain threshold; MDT, mechanical detection threshold; MPS, mechanical pain sensitivity; MPT, mechanical pain threshold; PPT, pressure pain threshold; TSL, thermal sensory limen; VDT, vibration detection threshold; WDT, warm detection threshold; WUR, wind‐up ratio. PHS and DMA not shown. *p = 0.016.

In the overall patient population, mean *z*‐scores for all QST parameters remained within the normal range on both the symptomatic and asymptomatic sides. However, a loss of function was observed in parameters assessing thermal perception (CDT, WDT, TSL), whereas a gain of function emerged in those related to pain processing (CPT, HPT, PPT, MPT, MPS, and WUR). Parameters evaluating large Aβ fiber function (MDT and VDT) showed only minimal changes. None of the patients had PHS or DMA (Figure 1, Table 2).

**TABLE 2:** QST *z*‐scores in the whole patients' cohort with trigeminal neuralgia (*n* = 65).

	Unaffected side	Affected side
CDT	**−1.45 ± 1.23** *	**−1.93 ± 1.44** *
WDT	−1.13 ± 1.22	−1.28 ± 1.30
TSL	−0.88 ± 1.10	−0.76 ± 1.22
CPT	1.22 ± 1.06	1.16 ± 1.25
HPT	0.89 ± 1.45	0.89 ± 1.51
PPT	1.19 ± 1.85	1.17 ± 1.79
MPT	0.58 ± 1.76	0.60 ± 1.52
MPS	0.78 ± 1.25	0.84 ± 1.24
WUR	1.17 ± 1.49	1.26 ± 1.35
MDT	0.18 ± 1.64	0.26 ± 0.97
VDT	−0.64 ± 1.47	−0.53 ± 1.29
PHS	0	0
DMA	0	0

*Note:* Mean ± SD of the 13 items of the DFNS protocol of Quantitative sensory Testing in 65 patients with Trigeminal Neuralgia (TN), on the affected and unaffected side of the face. In bold, statistically significant effects.

Abbreviations: CDT, cold detection threshold; CPT, cold pain threshold; DMA, dynamic mechanical allodynia; HPT, heat pain threshold; MDT, mechanical detection threshold; MPS, mechanical pain sensitivity; MPT, mechanical pain threshold; PHS, paradoxical heat sensation; PPT, pressure pain threshold; TSL, thermal sensory limen; VDT, vibration detection threshold; WDT, warm detection threshold; WUR, wind‐up ratio.

* *p* = 0.016.

In the whole patient population, only the CDT was significantly lower on the affected side compared to the unaffected side (*p* = 0.016). When we analyzed QST parameters according to the etiology (i.e., classical vs. idiopathic TN), we found that CDT fell outside the normative range of 0 ± 1.96 in the affected side of patients with classical TN (*z*‐score of −2.12 ± 1.40 in the affected side versus *z*‐score of −1.44 ± 1.10 in the unaffected side, *p* = 0.009), but not in patients with idiopathic TN, in which no asymmetry was found (*z*‐score of −1.61 ± 1.49 in the affected side versus *z*‐score of −1.47 ± 1.44 in the unaffected side, *p* > 0.5, Table 3). We found also a significant effect of the etiology of TN on WUR (F1,116 = 20.86, *p* < 0.0001); post hoc analysis showed that WUR was higher in idiopathic TN on both affected (*p* = 0.040) and unaffected side (*p* = 0.002).

**TABLE 3:** QST *z*‐scores in classical and idiopathic trigeminal neuralgia.

	Classical TN (*n* = 41)		Idiopathic TN (*n* = 24)	
	Unaffected side	Affected side	Unaffected side	Affected side
CDT	**−1.44 ± 1.10** *	**−2.12 ± 1.40** *	−1.47 ± 1.44	−1.61 ± 1.49
WDT	−1.05 ± 1.16	−1.32 ± 1.25	−1.26 ± 1.33	−1.22 ± 1.41
TSL	−0.84 ± 1.02	−0.77 ± 1.02	−0.96 ± 1.24	−0.74 ± 1.52
CPT	1.31 ± 1.17	1.18 ± 1.27	1.05 ± 0.82	1.13 ± 1.24
HPT	1.04 ± 1.51	1.01 ± 1.56	0.63 ± 1.32	0.68 ± 1.43
PPT	1.38 ± 1.79	1.26 ± 1.68	0.85 ± 1.93	1.02 ± 1.99
MPT	0.60 ± 1.98	0.61 ± 1.55	0.55 ± 1.37	0.58 ± 1.51
MPS	0.77 ± 1.36	0.78 ± 1.30	0.79 ± 1.07	0.96 ± 1.16
WUR	**0.75 ± 1.34** **	**0.97 ± 1.23** **	**1.87 ± 1.49** **	**1.73 ± 1.42** **
MDT	0.28 ± 0.96	0.20 ± 1.14	0.02 ± 2.36	0.36 ± 0.64
VDT	−0.73 ± 1.59	−0.66 ± 1.39	−0.47 ± 1.25	−0.30 ± 1.07

*Note:* Mean ± SD for 11 items of the DFNS protocol of Quantitative sensory Testing in 41 patients with Classical Trigeminal Neuralgia (TN) and 24 patients with Idiopathic TN, on the affected and unaffected side of the face. In bold, statistically significant effects.

Abbreviations: CDT, cold detection threshold; CPT, cold pain threshold; HPT, heat pain threshold; MDT, mechanical detection threshold; MPS, mechanical pain sensitivity; MPT, mechanical pain threshold; PPT, pressure pain threshold; TSL, thermal sensory limen; VDT, vibration detection threshold; WDT, warm detection threshold; WUR, wind‐up ratio; PHS and DMA not shown.

* *p* = 0.009.

** *p* < 0.0001.

Moreover, a significant effect of the type of pain (i.e., paroxysmal pain only versus concomitant continuous pain) was found on HPT (F1,116 = 16.95, *p* < 0.0001); post hoc analyses showed that *z*‐scores for HPT were higher in patients with purely paroxysmal pain on both affected (*p* = 0.031) and unaffected side (*p* = 0.016) compared to patients suffering also from concomitant continuous pain (Table 4) (i.e., patients with paroxysmal pain had gain of function in this QST parameter).

**TABLE 4:** QST *z*‐scores in patients with purely paroxysmal and concomitant continuous pain.

	Purely paroxysmal pain (*n* = 35)		Concomitant continuous pain (*n* = 30)	
	Unaffected side	Affected side	Unaffected side	Affected side
CDT	−1.60 ± 1.06	−1.91 ± 1.23	−1.27 ± 1.40	−1.95 ± 1.68
WDT	−1.11 ± 1.22	−1.36 ± 1.11	−1.15 ± 1.24	−1.19 ± 1.51
TSL	−0.95 ± 0.88	−0.83 ± 0.95	−0.80 ± 1.32	−0.67 ± 1.49
CPT	1.45 ± 1.00	1.35 ± 1.33	0.95 ± 1.01	0.95 ± 1.13
HPT	**1.43 ± 1.39** *	**1.29 ± 1.51** *	**0.26 ± 1.27** *	**0.43 ± 1.40** *
PPT	0.87 ± 1.87	0.80 ± 1.87	1.59 ± 1.76	1.63 ± 1.59
MPT	0.89 ± 1.71	0.92 ± 1.51	0.23 ± 1.78	0.24 ± 1.47
MPS	0.93 ± 1.26	1.01 ± 1.17	0.61 ± 1.24	0.66 ± 1.31
WUR	1.10 ± 1.52	1.32 ± 1.45	1.26 ± 1.48	1.20 ± 1.24
MDT	0.56 ± 0.29	0.49 ± 0.63	−0.18 ± 2.22	0.05 ± 1.18
VDT	−0.66 ± 1.41	−0.60 ± 1.30	−0.61 ± 1.55	−0.44 ± 1.30

*Note:* Mean ± SD for 11 items of the DFNS protocol of quantitative sensory testing in 35 patients with purely paroxysmal trigeminal neuralgia and 30 patients with trigeminal neuralgia with concomitant continuous pain, on the affected and unaffected side of the face. In bold, statistically significant effects.

Abbreviations: CDT, cold detection threshold; CPT, cold pain threshold; HPT, heat pain threshold; MDT, mechanical detection threshold; MPS, mechanical pain sensitivity; MPT, mechanical pain threshold; PPT, pressure pain threshold; TSL, thermal sensory limen; VDT, vibration detection threshold; WDT, warm detection threshold; WUR, wind‐up ratio; PHS and DMA not shown.

* *p* < 0.0001.

No significant correlation was found between QST parameters on both sides and disease duration.

### 3.3 Sensory Phenotyping

Using a deterministic approach, 32 patients were allocated to mechanical hyperalgesia (49%) phenotype, 23 to thermal hyperalgesia (35%), 5 to sensory loss (8%), and 5 to healthy profile (8%). Phenotype allocation was not statistically different between classical or idiopathic TN or between patients with purely paroxysmal pain and those without concomitant continuous pain also, or between patients taking or not sodium‐channel blockers. However, among patients taking sodium‐channel blockers, sensory phenotyping was significantly different in those with an unsatisfactory global pain relief, with higher frequency of sensory loss (*p* = 0.026) (Table 5).

**TABLE 5:** Sensory phenotype allocation.

	Sensory loss	Thermal hyperalgesia	Mechanical hyperalgesia	Healthy
All patients	5 (8%)	23 (35%)	32 (49%)	5 (8%)
Classical TN	3 (7.5%)	15 (37.5%)	19 (47.5%)	3 (7.5%)
Idiopathic TN	2 (8%)	8 (32%)	13 (52%)	2 (8%)
Purely paroxysmal	2 (6%)	11 (31%)	20 (57%)	2 (6%)
Concomitant continuos pain	3 (10%)	12 (40%)	12 (40%)	3 (10%)
With sodium channel blockers	3 (7.5%)	15 (37.5%)	18 (45%)	4 (10%)
Without sodium channel blockers	2 (8%)	8 (32%)	14 (56%)	1 (4%)
Responder to sodium channel blockers*	**0**	**11 (37%)**	**15 (50%)**	**4 (13%)**
Non responder to sodium channel blockers*	**3 (30%)**	**4 (40%)**	**3 (30%)**	**0**

*Note:* Sensory phenotype allocation of the whole patients' cohort and of the subgroup of interest, using a deterministic approach. In bold, statistically significant different allocation between the specific subgroups.

* *p* = 0.026.

## 4 Discussion

In this cross‐sectional study in patients with TN we found a loss of function in QST parameters assessing thermal perception as well a gain of function in those related to pain processing, on both the symptomatic and asymptomatic sides. Etiology was significantly associated with QST parameters: unilateral altered CDT was specific to classical TN, while WUR was bilaterally increased in idiopathic TN; patients with paroxysmal pain had higher HPT on both sides. Sensory phenotyping showed that a sensory loss profile was associated with a poor response to sodium channel blockers.

### 4.1 Quantitative Sensory Testing

Unlike previous single‐center studies with a smaller patient population [15, 16], we are the first to apply the full battery of 13 DFNS QST tests to a relatively large cohort of patients with TN. This enabled us to uncover previously unreported aspects of somatosensory function in TN. Our findings indicate that somatosensory processing in TN is marked by complex and subtle alterations affecting both the affected and unaffected sides of the face. Specifically, we observed a bilateral loss of function in thermal perception and an increased sensitivity in pain‐related modalities; the latter consistent with findings from a previous study [15]. Only minimal changes in mechanical detection and vibration thresholds (MDT and VDT) were found. The symmetrical, bilateral distribution of abnormalities involving thermal‐pain sensory function—including involvement of the non‐painful side—may point to centrally mediated modulation of sensory processing, as already evidenced in many other conditions of unilateral peripheral neuropathic pain [17, 18, 19].

Interestingly, our study did not show significant abnormalities in VDT and MDT—Aβ‐related sensory modalities—despite the anticipated involvement of these fibers in TN pathology [20]. Previous studies [15, 16] found impaired MDT in patients with TN, an observation not replicated by our study. This discrepancy may be attributed to a floor effect in MDT assessment observed in our patient sample. Most patients had MDT values corresponding to the lowest detectable von Frey filament force, possibly due to the uniquely high density of sensory innervation and low‐threshold mechanoreceptors in the facial region [13]. A possible explanation for the absence of significant abnormalities in VDT is that the assessment may be affected by contralateral transmission of vibration through the skull bones, thereby reducing its reliability in detecting unilateral sensory deficits [21].

Although our patients showed thermal loss of function, none experienced PHS during the TSL procedure. PHS are commonly reported in conditions such as acute CRPS type I [19], where they may be driven by inflammatory mechanisms, and in multiple sclerosis [22], where they are thought to reflect central supraspinal alterations—mechanisms not considered central to TN. Because PHS have been hypothesized to arise from central disinhibition of polymodal second‐order neurons due to reduced input from cold‐specific afferents [22], we speculate that the high density of sensory innervation in the facial region may exert a protective effect, making it more difficult to reach the disinhibition threshold even in the presence of sensory loss. Consistent with this interpretation, no PHS were reported in the subgroup of patients with TN within the DFNS neuropathic pain cohort [23].

When we analyzed QST parameters by etiology (classical vs. idiopathic TN), we found that only patients with classical TN had a significant asymmetry in CDT between the affected and unaffected sides. A previous study also reported a slight CDT asymmetry [16]; however, possibly due to a limited sample size, statistical significance was reached only in patients with V2 involvement. This side asymmetry in the CDT is compatible with a specific damage to somatosensory Aδ afferents in classical TN, possibly due to neurovascular compression. The Aδ fibers are small, myelinated fibers responsible for transmitting noxious or cold stimuli. Histopathological studies have revealed dysmelination of primary trigeminal afferents in TN [20], a mechanism thought to cause hyperexcitability, ectopic excitation, ephaptic transmission, and high frequency discharges, all of which may underlie the paroxysmal pain attacks characteristic of the condition [24]. Although most commonly reported triggers in TN are linked to light tactile stimuli, mediated by Aß fibers [25], the specific role of Aδ fiber in TN pathophysiology has been recently highlighted by the observation that their disfunction may underlie some of the less reported triggers, like cold wind, cold food, and even specific food triggers through an osmotic mechanism [26].

Consistent with the analysis by etiology, we also found that idiopathic TN was associated with significantly higher bilateral WUR, a QST parameter linked to the temporal summation of nociceptive stimuli and considered a sign of second‐order neuron hyperexcitability [17]. Because the increase in WUR was bilateral, the side‐to‐side difference remained small and was not detected in the side‐to‐side comparison analysis. We hypothesize that this central hyperexcitability may act as a sensitizing predisposition, allowing otherwise minor unilateral damage to peripheral trigeminal afferents—such as simple neurovascular contact without clear radiological evidence of root pathology—to contribute to triggering paroxysmal pain attacks. This hypothesis could help explain the clinical efficacy of microvascular decompression surgery even in cases of idiopathic TN [27].

In terms of type of pain, patients with purely paroxysmal pain had significantly increased bilateral HPT compared to patients with concomitant continuous pain, with similar (though nonsignificant) trends observed in other pain modalities. Because the increase in HPT was bilateral, the side‐to‐side difference remained small and was not detected in the side‐to‐side comparison analysis. This difference may reflect distinct mechanisms of central sensitization in patients with or without concomitant continuous pain. One possible explanation is that patients with concomitant continuous pain experience a sustained activation of endogenous pain inhibitory systems, which may not be similarly engaged by the episodic pain seen in purely paroxysmal TN [28].

In our patients with TN, none of the abnormalities in QST parameters, including bilateral changes, correlated with disease duration, unlike what has been reported in other conditions such as CRPS I [19]. These findings are consistent with a previous study in patients with TN showing that trigeminal root atrophy does not correlate with disease duration [3]. Taken together, these observations suggest that in TN, somatosensory system abnormalities develop early in the disease course and remain relatively stable over time.

### 4.2 Sensory Phenotyping

In our study, we provide previously unreported information on sensory phenotyping in patients with TN. The most common phenotype we observed was mechanical hyperalgesia, which is presumed to be associated with central sensitization, followed by thermal hyperalgesia, believed to reflect peripheral receptor hyperactivity [11]. These profiles are thought to predict responsiveness to drugs commonly used in neuropathic pain treatment, such as oxcarbazepine [11]. Notably, in our cohort, patients with an unsatisfactory response to sodium channel blockers had a significantly different sensory phenotype compared to responders, with a higher frequency of sensory loss. Consistently, none of the patients who showed a brilliant response to these medications presented with a sensory loss phenotype. While the low overall prevalence of the sensory loss phenotype in our population prevents firm conclusions, the observed pattern may suggest the involvement in these patients of additional pathophysiological mechanisms, linked to peripheral deafferentation, which are not adequately targeted by sodium channel blockers. These findings align with a previous study demonstrating that oxcarbazepine treatment is more effective in patients with painful neuropathy with an “irritable nociceptors” phenotype [29]. Future studies should investigate whether sensory phenotypes can predict differential responses to surgical therapy, thereby supporting earlier referral for patients with phenotypes potentially less responsive to medical treatment.

### 4.3 Limitations

To ensure that the most affected division was correctly assessed, the examiner performing QST was not blinded to pain localization. Although blinding is not explicitly required by the DFNS protocol, this may still represent a potential source of bias. However, we consider this risk to be minimal given our strict adherence to the DFNS procedures, which emphasize protocol standardization as the primary mechanism to reduce examiner‐related bias.

Admittedly we did not perform QST assessment on non‐trigeminal regions; therefore, based on our data, we cannot determine whether sensory abnormalities in patients with TN extend beyond the trigeminal territory, although previous evidence exists on this topic [15]. Given the considerable time required to complete the entire testing protocol, performing assessments on an additional non‐trigeminal site within a single session could have negatively impacted patient cooperation, which remains crucial for the reliability of QST results.

## 5 Conclusions

Our study, which identified specific QST abnormalities in patients with TN, demonstrates that this tool may enhance our understanding of this condition, including its etiology, type of pain, and treatment response. QST showed bilateral thermal hypoesthesia and pain hyperesthesia across the entire patient group.

CDT was significantly reduced on the affected side in patients with Classical TN, while Idiopathic TN was associated with bilaterally elevated WUR. Patients with purely paroxysmal pain showed a bilateral gain of function in HPT.

Admittedly, the nature of these sensory abnormalities likely limits the clinical usefulness of QST for routine diagnostic use. This highlights the need for novel methods to assess somatosensory function in the facial region, with the aim of supporting the integration of TN into the diagnostic grading system for neuropathic pain conditions [7, 8].

## Author Contributions

**Gianfranco De Stefano:** conceptualization, methodology, data curation, investigation, formal analysis, funding acquisition, visualization, project administration, writing – original draft, writing – review and editing. **Daniel Litewczuk:** data curation, investigation, formal analysis, writing – original draft. **Eleonora Galosi:** methodology, data curation, formal analysis. **Giuseppe Di Pietro:** data curation, investigation, formal analysis. **Pietro Falco:** methodology, data curation, formal analysis. **Caterina Leone:** methodology, data curation, supervision. **Jibril Osman‐Farah:** methodology, investigation, supervision, project administration. **Deepti Bhargava:** investigation, supervision. **Francis O'Neill:** methodology, investigation, supervision, project administration, writing – review and editing. **Bernhard Frank:** methodology, investigation, supervision, project administration, resources, writing – review and editing. **Colby T. Joncas:** writing – review and editing. **Raymond F. Sekula Jr.:** writing – review and editing. **Giulia Di Stefano:** conceptualization, methodology, data curation, formal analysis, supervision, funding acquisition, writing – review and editing. **Andrea Truini:** conceptualization, methodology, data curation, formal analysis, supervision, resources, writing – review and editing.

## Funding

This work was supported by European Academy of Neurology through EAN Research Training Fellowship 2021 and by the International Association for the Study of Pain through IASP Collaborative Research Grant 2021.

## Conflicts of Interest

The authors declare no conflicts of interest.

## Data Availability

The data that support the findings of this study are available on request from the corresponding author. The data are not publicly available due to privacy or ethical restrictions.
